# Progestin-only pretreatment enhances follicular synchronization and embryo development: a three-arm retrospective cohort study in GnRH antagonist cycles

**DOI:** 10.1186/s40834-025-00396-x

**Published:** 2025-09-24

**Authors:** Masato Kobanawa

**Affiliations:** Kobanawa Clinic, 169-3 Tagiya, Omitama-shi, Ibaraki Japan

**Keywords:** Progestin-only pretreatment, Combined oral contraceptive pills, Follicular synchronization, In vitro fertilization, Controlled ovarian stimulation, Cumulative live birth rate

## Abstract

**Background:**

Hormonal pretreatment prior to controlled ovarian stimulation (COS) in assisted reproductive technology (ART) is widely used to improve follicular synchronization and schedule flexibility. However, the comparative impact of different pretreatment strategies—progestin-only, combined oral contraceptive pills (COCP), and no pretreatment—on follicular dynamics and clinical outcomes remains unclear.

**Methods:**

In this retrospective cohort study, 240 patients undergoing their first IVF/ICSI cycle with a GnRH antagonist protocol and recombinant FSH monotherapy were analyzed. Participants were divided into three groups: progestin-only pretreatment (P group), COCP pretreatment (COCP group), and no pretreatment (control group). Baseline characteristics, follicular development, embryo quality, and cumulative reproductive outcomes were compared. Levene’s test was used to assess follicular size variability, and multivariable regression analyses were performed to adjust for confounding variables.

**Results:**

The P group demonstrated significantly improved follicular synchronization, as evidenced by the lowest variability in the ratio of follicles ≥ 18 mm to ≥ 14 mm. Embryological outcomes were superior in the P group, with higher oocyte maturation rate (*p* = 0.040), fertilization rate (*p* = 0.038), and number of good-quality blastocysts (*p* = 0.015) compared to the other groups. The blastocyst development rate was also significantly greater (*p* = 0.029). Although cumulative live birth rate (CLBR) did not reach statistical significance, a trend toward higher CLBR was observed in the P group (87.0%) compared to COCP (72.0%) and control (75.2%).

**Conclusion:**

Progestin-only pretreatment leads to superior follicular synchronization and improved embryo developmental potential in GnRH antagonist IVF/ICSI cycles. These benefits may enhance ART efficiency and contribute to improved cumulative outcomes.

## Introduction

Hormonal contraceptives have a broad range of indications beyond pregnancy prevention in the field of gynecology. Their use as pretreatment prior to assisted reproductive technologies (ART), particularly in controlled ovarian stimulation (COS), includes menstrual cycle scheduling, synchronization of the oocyte cohort, modulation of the endocrine environment prior to stimulation, and prevention of functional ovarian cyst formation [1].

The suppressive effects of hormonal contraceptives on follicle-stimulating hormone (FSH) and luteinizing hormone (LH) may facilitate better synchronization of follicular development during COS. Combined oral contraceptive pills (COCPs), which include both estrogen and progestin, strongly suppress the hypothalamic–pituitary–ovarian (HPO) axis. However, concerns have been raised regarding their potential negative impact on follicular growth and endometrial receptivity. In contrast, progestin-only agents (e.g., norethisterone) may suppress GnRH pulsatility with relatively less interference in endogenous hormonal dynamics [2].

A prospective randomized trial investigating hormonal pretreatment prior to in vitro fertilization (IVF) found that women who received hormonal contraceptives required higher total gonadotropin doses, but this did not adversely affect the number of oocytes retrieved or pregnancy outcomes [3]. Furthermore, a meta-analysis of four randomized controlled trials (RCTs) concluded that, in GnRH antagonist cycles, women with and without prior hormonal contraceptive pretreatment had comparable ongoing pregnancy rates and oocyte retrieval outcomes [4].

Thus, the effectiveness of pretreatment prior to COS initiation remains unclear. In particular, few studies have comprehensively compared the effects of COCPs, progestin-only agents, and no pretreatment on hormonal profiles, follicular dynamics, and clinical outcomes—including cumulative pregnancy and live birth rates—in women with normal ovarian response.

Therefore, the objective of this study was to investigate whether hormonal pretreatment strategies—including progestin-only agents and combined oral contraceptive pills—have an impact on ART outcomes, by comparing hormone profiles, follicular development, and cumulative reproductive results in GnRH antagonist IVF/ICSI cycles.

## Materials and methods

### Study population and design

This retrospective cohort study was conducted at Kobanawa Clinic and included 240 ART cycles performed between April 2022 and December 2024. A total of 363 cases were initially screened, and 240 cycles meeting the eligibility criteria were included after applying exclusion criteria. After approval by the Medical Corporation Kobanawa Clinic Ethic Screening Committee, this study was conducted with opt-out disclosure of information.

Based on previous studies reporting cumulative live birth rates (CLBR) ranging from 76–86% [5], sample size calculation was performed using Cohen’s *w* for chi-square testing among three independent groups. The resulting effect size was estimated as *w* ≈ 0.26 (small to medium) [6].

Using G*Power version X.X (Heinrich Heine University, Düsseldorf, Germany), with a two-sided significance level of 0.05 and 80% power, the minimum required sample size was calculated to be 108 participants (36 per group) [7].

This study included the first ART cycle of each patient who underwent controlled ovarian stimulation (COS) using recombinant FSH (rFSH) monotherapy with either follitropin alfa, beta, or delta. All patients were covered under the Japanese Healthcare Insurance System and were aged ≤ 42 years.

**Fig. 1:**
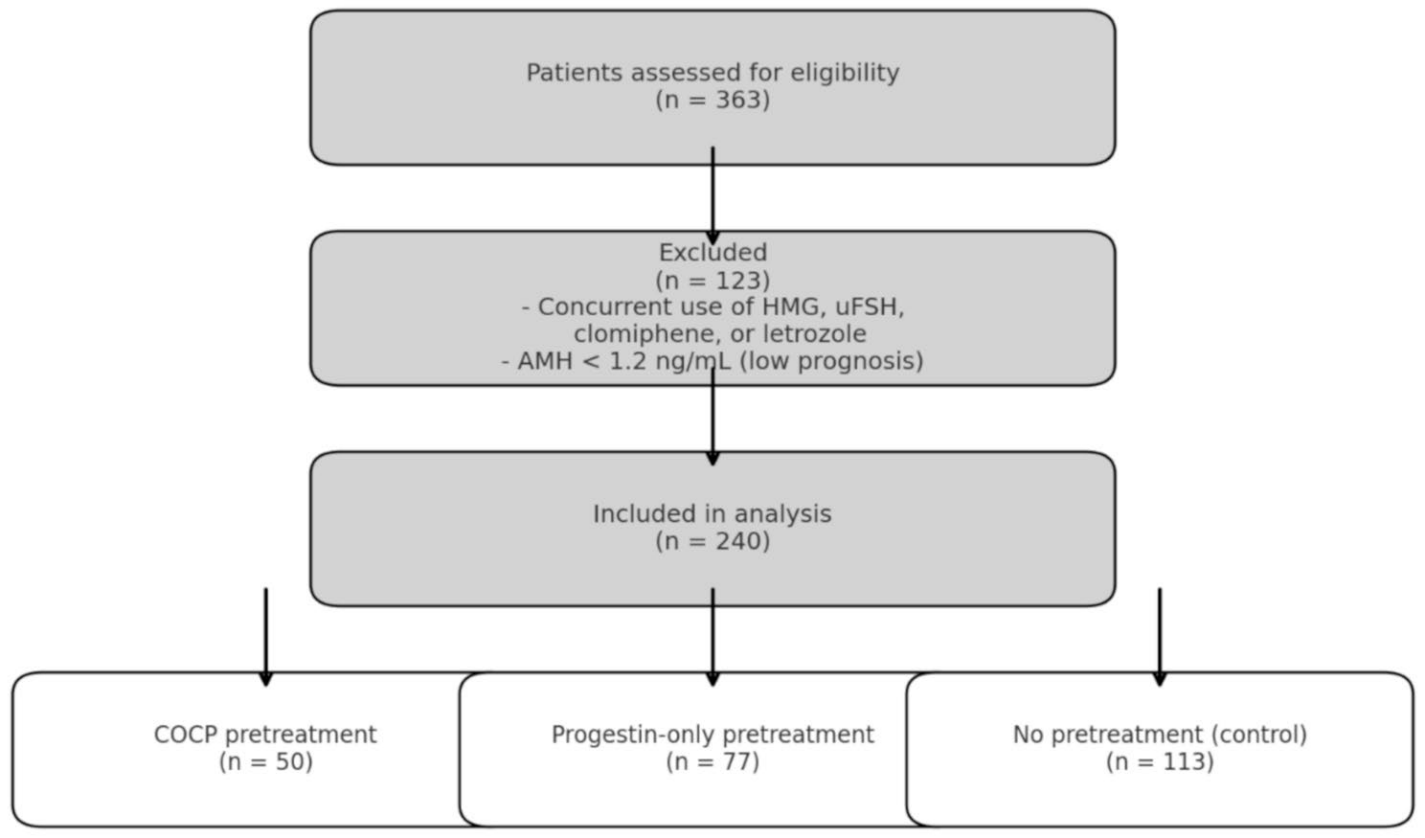
Among 363 patients assessed for eligibility, 123 were excluded due to (1) concurrent use of HMG, urinary FSH, clomiphene, or letrozole, or (2) low ovarian reserve, defined as AMH < 1.2 ng/mL A total of 240 patients were included in the final analysis They were categorized into three groups based on pretreatment: COCP pretreatment (*n* = 50), progestin-only pretreatment (P group, *n* = 77), and no pretreatment (control group, *n* = 113)

Exclusion criteria included patients who deviated from the prescription guidelines for Follitropin Alfa, Beta and Delta, those who received concurrent concomitant treatment with human menopausal gonadotropin (HMG), urinary FSH (uFSH), clomiphene, or letrozole, and those who had anti-Müllerian hormone (AMH) levels < 1.2 ng/mL, classified as low prognosis (Fig. 1).

This retrospective study included patients who underwent controlled ovarian stimulation (COS) with or without pretreatment using combined oral contraceptive pills (COCP) or progestin-only pretreatment (P). In the COCP and P groups, pretreatment medications were administered for a variable number of days. The duration was determined based on patient preference and clinic scheduling needs.

The choice of pretreatment was not randomized, but rather based on clinical discretion and patient preference as part of routine care.

### Controlled ovarian stimulation and oocyte retrieval

In COS, daily subcutaneous injections of Follitropin Alfa (Gonal F; Merck BioPharma, Tokyo, Japan) or Follitropin Beta (Foristim, Organon, Osaka, Japan) or Follitropin Delta (Rekovelle; Ferring Pharma, Tokyo) as a mono protocol with rFSH were administered starting on days 1–3 of menstruation, using the COS with GnRH antagonist protocol.

The starting dose of follitropin alfa and beta was determined using the gonadotropin starting dose calculator developed by Kobanawa with a fixed dose [8].

The daily individualized dose of follitropin delta was determined using the serum anti-Müllerian hormone (AMH) level within the previous 12 months and body weight, with a fixed dose used throughout the stimulation [9].

Gonadotropin-releasing hormone antagonists (Ganirest; MSD, Tokyo, Japan) were started at a dose of 0.25 mg/day when the primary follicle reached approximately 14 mm. When several leading follicles reached 17–20 mm, gonadotropins and GnRH antagonist doses of 0.25 mg/day were terminated, and on the same day or the next day, 250 µg of choriogonadotropin alfa was administered.

Oocyte retrieval was performed 36–39 h after triggering.

### Fertilization, embryo culture, and embryo transfer

After oocyte retrieval, insemination and intracytoplasmic sperm injection (ICSI) were performed.

Then, fertilized oocytes were cultured to blastocysts, which were then frozen.

In accordance with routine Japanese ART practice under the national insurance system, all embryos were cultured to the blastocyst stage before cryopreservation and transfer, as this approach improves pregnancy outcomes and also increases reimbursement points, ensuring cost-effectiveness.

Thawed embryos were transferred during the next menstrual cycle or later by hormone replacement cycles (HRCs) or natural cycles (NCs). In HRC, hormone replacement of estrogen (Estrana Tapes, Hisamitsu Pharmaceutical, Tokyo) at 0.72 mg ×4 every other day was started on days 1–3 of menstruation. With endometrial thickening confirmed to be at least 7 mm, progesterone (UTROGESTAN; Fuji Pharma Co.,Ltd., Tokyo) 200 mg ×3/day was commenced, and blastocyst transfer was performed six days later (*P* + 5). We performed a single embryo transfer (SET). In the NC group, blastocysts were transferred on the fifth day after natural ovulation without the use of drugs.

Clinical pregnancy was defined as a case in which the fetal sac was confirmed by transvaginal ultrasonography within four to five weeks of gestation determined from the day of embryo transfer. After pregnancy, birth outcomes were tracked based on reports from the patients or the hospitals where the delivery occurred.

### Hormonal and laboratory assessments

Blood samples were collected during the study to assess AMH, FSH, luteinizing hormone (LH), estradiol, and progesterone levels. AMH concentrations were measured during screening before the start of the cycle and used to determine the starting dose of gonadotropins. AMH levels were measured using an automated Elecsys AMH assay (Roche Diagnostics, Basel, Switzerland). Serum samples were used to assess endocrine parameters (FSH, LH, estradiol, and progesterone).

### Outcome measures

Mature oocytes (MII) were defined as oocytes confirmed by denudation for intracytoplasmic sperm injection (ICSI) or oocytes confirmed zygotes with two pronuclei by insemination (conventional IVF).

The fertilization rate was defined as the number of pronuclei confirmed per insemination by IVF or ICSI punctures. The embryo culture results were compared based on blastocyst rates, defined as the number of high-quality blastocysts (Gardner’s classification of 3BB or higher) per cultured embryo [10].

Clinical pregnancy was defined as a case in which the fetal sac was confirmed by transvaginal ultrasonography within four to five weeks of gestation determined from the day of embryo transfer. After pregnancy, birth outcomes were tracked based on reports from the patients or the hospitals where the delivery occurred.

Cumulative pregnancy and live birth rates were calculated as the probability of a single-cycle embryo leading to pregnancy or live birth over multiple transfers [11].

As the primary purpose of this study was to compare cumulative results per stimulation cycle across pretreatment groups, the cumulative live birth rate (CLBR) was calculated using a conservative per-cycle approach. This included: cycles in which at least one pregnancy and delivery occurred after multiple embryo transfers using frozen-thawed embryos derived from a single COS cycle; cycles in which pregnancy did not occur even after transfer of all available embryos; and cycles in which embryo freezing was unsuccessful due to poor embryo development or quality. All eligible stimulation cycles that were initiated between April 2022 and December 2024 were included, and each cycle was followed until either a live birth achieved or all embryos had been exhausted, with follow-up completed by March 2025. Notably, no patients discontinued treatment during the study period; therefore, all cycles could be conclusively classified as either “live birth” or “no live birth.”

In order to confirm the robustness of the findings, we conducted a sensitivity analysis restricted to patients who underwent at least one embryo transfer. Cycles in which no embryo transfer occurred (due to fertilization failure, poor embryo development, or lack of viable embryos for freezing) were excluded from this analysis. CLBR was re-evaluated in this subpopulation to assess whether the trends remained consistent across pretreatment groups.

### Statistical analysis

For baseline patient characteristics, numerical variables were compared among the three groups using one-way analysis of variance (ANOVA), while categorical variables were compared using the chi-square test.

To assess clinical outcomes, multivariable analyses were conducted to adjust for potential confounders identified as significantly different among the groups at baseline. Specifically, multiple linear regression and logistic regression analyses were performed, incorporating these covariates to compare the three groups.

To evaluate the variability in follicular size synchronization, Levene’s test was used to compare the homogeneity of variance among groups.

All statistical analyses were performed using EZR (Saitama Medical Center, Jichi Medical University, Saitama, Japan), which is a graphical user interface for R (The R Foundation for Statistical Computing, Vienna, Austria). EZR is a modified version of the R Commander designed to provide additional statistical functions frequently used in biostatistics [12].

## Results

Baseline characteristics were compared among the three groups: COCP, Control, and Progesterone (P) (Table 1).

**Table 1:** Baseline characteristics and treatment parameters of the three groups

Variable	COCP	Control	Progestin	*p*-value
*n*	50	113	77	
Duration of medication (days)	13.52 (3.25)	0 (0)	9.87 (0.75)	< 0.001
Age, years	32.82 (3.90)	34.39 (3.68)	32.79 (3.90)	0.006
AMH, ng/ml	4.45 (3.06)	4.17 (2.70)	4.37 (2.80)	0.814
AFC, follicles	15.56 (6.43)	16.41 (6.38)	17.17 (7.11)	0.407
Basal E2, pg/ml	35.53 (21.36)	67.65 (392.89)	25.34 (16.09)	0.538
Basal FSH, IU/L	7.60 (2.17)	6.97 (1.61)	6.68 (1.70)	0.017
Basal LH, IU/L	7.88 (3.40)	6.02 (2.22)	6.37 (3.03)	< 0.001
Body weight, kg	64.97 (53.53)	57.62 (10.99)	58.89 (11.99)	0.253
Gonadotropin.preparation: Follitropin alfa, *n* (%)	27 (54.0)	58 (51.3)	43 (55.8)	0.97
Gonadotropin.preparation: Follitropin beta, *n* (%)	2 (4.0)	6 (5.3)	3 (3.9)	
Gonadotropin.preparation: Follitropin delta, *n* (%)	21 (42.0)	49 (43.4)	31 (40.3)	
Trigger.methods: GnRH, *n* (%)	47 (94.0)	94 (83.2)	65 (84.4)	0.172
Trigger.methods: hCG, *n* (%)	3 (6.0)	19 (16.8)	12 (15.6)	
Fertilization.methods: c-IVF, *n* (%)	44 (88.0)	78 (69.0)	65 (84.4)	0.007
Fertilization.methods: ICSI, *n* (%)	6 (12.0)	35 (31.0)	12 (15.6)	

Numerical variables are presented as mean (standard deviation), and categorical variables as number (percentage). Statistical comparisons among the COCP, Control, and Progesterone groups were performed using one-way ANOVA for numerical variables and chi-square tests for categorical variables

There were no missing data for baseline characteristics and outcome measures in this study.

There were significant differences in patient age (*p* = 0.006), basal FSH (*p* = 0.017), and basal LH levels (*p* < 0.001) among the groups. The duration of medication also varied significantly, with the COCP group having the longest mean duration (13.52 ± 3.25 days) and the Control group having none due to lack of pretreatment (*p* < 0.001). However, there was no significant difference in duration between the COCP and P groups, suggesting comparable exposure to pretreatment medications within those two groups.

The proportion of fertilization methods differed significantly among groups (*p* = 0.007), with the COCP and Progesterone groups showing a higher rate of conventional IVF compared to the Control group. Other variables, including AMH, AFC, basal estradiol, body weight, gonadotropin preparation type, and trigger method, did not show statistically significant differences between groups (Table 1).

Based on these results, age, basal FSH, basal LH, and fertilization method were considered potential confounding factors and were included as covariates in subsequent multivariable analyses.

Compared to the COCP and control groups, the P group demonstrated significantly higher values in several key embryological outcomes. Specifically, the oocyte maturation rate (*p* = 0.040), fertilization rate (*p* = 0.038), and the number of good-quality blastocysts (*p* = 0.015) were all significantly greater in the P group after adjustment for confounding factors. In addition, the blastocyst development rate was also significantly improved (*p* = 0.029) (Table 2).

**Table 2:** Comparison of ovarian stimulation characteristics, embryological outcomes, and clinical outcomes among the COCP, Control, and P groups

Variable	COCP	Control	Progestin	Univariate *P*-value	Multivariate *P*-value
Stimulation days, days	13.54 (2.13)	13.49 (3.00)	14.39 (2.97)	0.079	0.078
Maximum follicle diameter on days 6–8 of stimulation, mm	16.7 (3.77)	16.05 (3.09)	16.39 (3.55)	0.51	0.513
minimum follicle diameter on days 6–8 of stimulation, mm	9.12 (1.76)	9.57 (2.08)	9.22 (2.67)	0.398	0.256
Follicle count ≥ 14 mm on the day of trigger, folllicles	16.96 (6.04)	17 (6.51)	17.47 (7.52)	0.876	0.780
Follicle count ≥ 16 mm on the day of trigger, folllicles	14.3 (5.23)	15.15 (6.08)	15.03 (7.18)	0.72	0.359
Follicle count ≥ 18 mm on the day of trigger, folllicles	10.44 (4.57)	11.26 (5.25)	12.34 (5.87)	0.132	0.197
Follicle count ≥ 20 mm on the day of trigger, folllicles	6.24 (4.19)	6.45 (4.78)	6.58 (4.93)	0.922	0.899
Total gonadotropin dosage, mcg	141.86 (57.35)	148.89 (74.10)	135.55 (51.57)	0.37	0.890
E2 level on the day of trigger, pg/ml	3880.98 (1615.67)	3889.8 (2076.56)	3994.14 (2606.07)	0.939	0.853
P4 level on the day of trigger, ng/ml	1.67 (2.38)	1.43 (0.82)	1.28 (0.93)	0.278	0.342
Number of oocytes retrieved, oocytes	14.94 (6.02)	16.03 (6.66)	16.43 (7.21)	0.466	0.736
Follicular Output Rate (FORT), No. follicles ≧ 16 mm /AFC	1.00 (0.42)	0.98 (0.38)	0.96 (0.50)	0.82	0.712
Follicle-to-Oocyte Index (FOI), No.oocytes/ AFC	0.98 (0.14)	0.97 (0.12)	0.95 (0.12)	0.487	0.470
Ovarian Sensitivity Index (OSI), oocytes/mcg	0.12 (0.06)	0.14 (0.10)	0.15 (0.14)	0.301	0.453
Number of mature oocytes, oocytes	14.56 (6.00)	15.36 (6.71)	16.16 (7.20)	0.421	0.803
Oocyte maturation rate, %	97.00 (6.00)	95.00 (8.00)	98.00 (5.00)	0.022	0.040
Number of two-pronuclei (2PN) zygotes, zygotes	8.38 (4.15)	10.05 (5.43)	10.66 (5.07)	0.044	0.160
Fertilization rate, %	58.00 (20.00)	66.00 (21.00)	67.00 (15.00)	0.028	0.038
Number of good-quality blastocysts, blastocysts	4.92 (3.10)	6.00 (4.02)	7.23 (4.22)	0.005	0.015
Blastocyst development rate, %	57.00 (22.00)	59.00 (22.00)	66.00 (21.00)	0.039	0.029
Number of embryo transfers, transfers	1.84 (1.28)	1.57 (0.99)	1.51 (0.88)	0.177	0.202
Cumulative pregnancy rate, % (n)	86 (43)	85 (96)	97.4(75)	0.01	0.090
Cumulative live birth rate, % (n)	72 (36)	75.2 (85)	87 (67)	0.0726	0.237

Data are presented as mean (standard deviation) for continuous variables or number (percentage) for categorical variables. Univariate *p*-values were calculated using one-way ANOVA or chi-square tests, as appropriate. For multivariable analysis, age, basal FSH, and basal LH were included as covariates for outcomes up to oocyte retrieval. For outcomes following fertilization, fertilization method was additionally included as a confounding factor. Age, which showed a significant difference among groups at baseline, was also incorporated as a covariate in all multivariable models to adjust for its potential confounding effect on embryological and clinical outcomes

Furthermore, although not reaching statistical significance in the multivariable analysis, the P group showed a trend toward a higher cumulative pregnancy rate (97.4%) compared with the COCP (86.0%) and control groups (85.0%) (*p* = 0.090), whereas no significant difference was observed in the cumulative live birth rate (CLBR) among the groups. (Table 2).

A subgroup analysis was performed among the 236 patients who underwent at least one embryo transfer. The cumulative live birth rate remained highest in the progestin group (88.2%), followed by the control group (75.9%) and the COCP group (75.0%).

Although the difference among the three groups did not reach statistical significance, a favorable trend was observed (*p* = 0.0816), consistent with the findings from the primary analysis (Table 3).

**Table 3:** Cumulative live birth outcomes among patients who underwent embryo transfer (*n* = 236)

Group	Live Birth, *n* (%)	No Live Birth, *n* (%)	*p*-value
COCP *n* = 48	36 (75.0)	12 (25.0)	
Control Group *n* = 112	85 (75.9)	27 (24.1)	
Progestin Group *n* = 76	**67 (88.2)**	**9 (11.8)**	**0.0816**

In addition, the effect of pretreatment on follicular synchronization was evaluated by comparing follicular size distribution among the three groups.

To assess the consistency of follicular synchronization patterns across different pretreatment groups, we evaluated the within-group variability of the proportions of follicles ≥ 20 mm, ≥ 18 mm, and ≥ 16 mm relative to follicles ≥ 14 mm.

Three indices of dispersion were calculated within each group: standard deviation (SD), variance (Var), and interquartile range (IQR). Among the three groups, the P group consistently showed the smallest values for all three dispersion measures (Table 4).

**Table 4:** Within-group variability in follicle size ratios (≥ 20 mm, ≥ 18 mm, ≥ 16 mm relative to ≥ 14 mm) across pretreatment groups

Ratio of follicles (≥ mm / ≥14 mm)	Measure	Progestin	COCP	Control
20 / 14	SD	0.23	0.25	0.24
	Var	0.05	0.06	0.06
	IQR	0.31	0.34	0.34
18 / 14	SD	0.22	0.25	0.19
	Var	0.05	0.06	0.04
	IQR	0.30	0.33	0.26
16 / 14	SD	0.14	0.15	0.13
	Var	0.02	0.02	0.02
	IQR	0.23	0.25	0.20

To formally assess whether the P group had significantly lower variability compared to the COCP and control groups, levene’s test was used to compare intra-group variability in follicle size synchronization, as measured by the ratios of follicles ≥ 20 mm, ≥ 18 mm, and ≥ 16 mm to those ≥ 14 mm. The analysis revealed that only the 18 mm/14 mm ratio showed a statistically significant difference in variance across pretreatment groups (*p* = 0.0001), while the 20 mm/14 mm and 16 mm/14 mm ratios did not (*p* = 0.4809 and *p* = 0.3710, respectively) (Table 5).

**Table 5:** Results of Levene’s tests comparing variance in follicle size synchronization ratios among the three pretreatment groups

Ratio of follicles (≥ mm / ≥14 mm)	Overall Levene Test *p*-value	Pairwise Comparison	Levene Statistic	*p*-value
20 / 14	0.4809	-	-	-
18 / 14	0.0001	Control vs. Progestin	5.353	0.0218
		Control vs. COCP	6.606	0.0111
		Progestin vs. COCP	18.947	< 0.0001
16 / 14	0.3710	-	-	-

To further explore this difference, post hoc pairwise Levene’s tests were performed for the 18 mm/14 mm ratio. The P group showed significantly lower variance than both the Control (*p* = 0.0218) and COCP groups (*p* < 0.0001), while the Control group also had significantly lower variance than the COCP group (*p* = 0.0111) (Table 4).

**Fig. 2:**
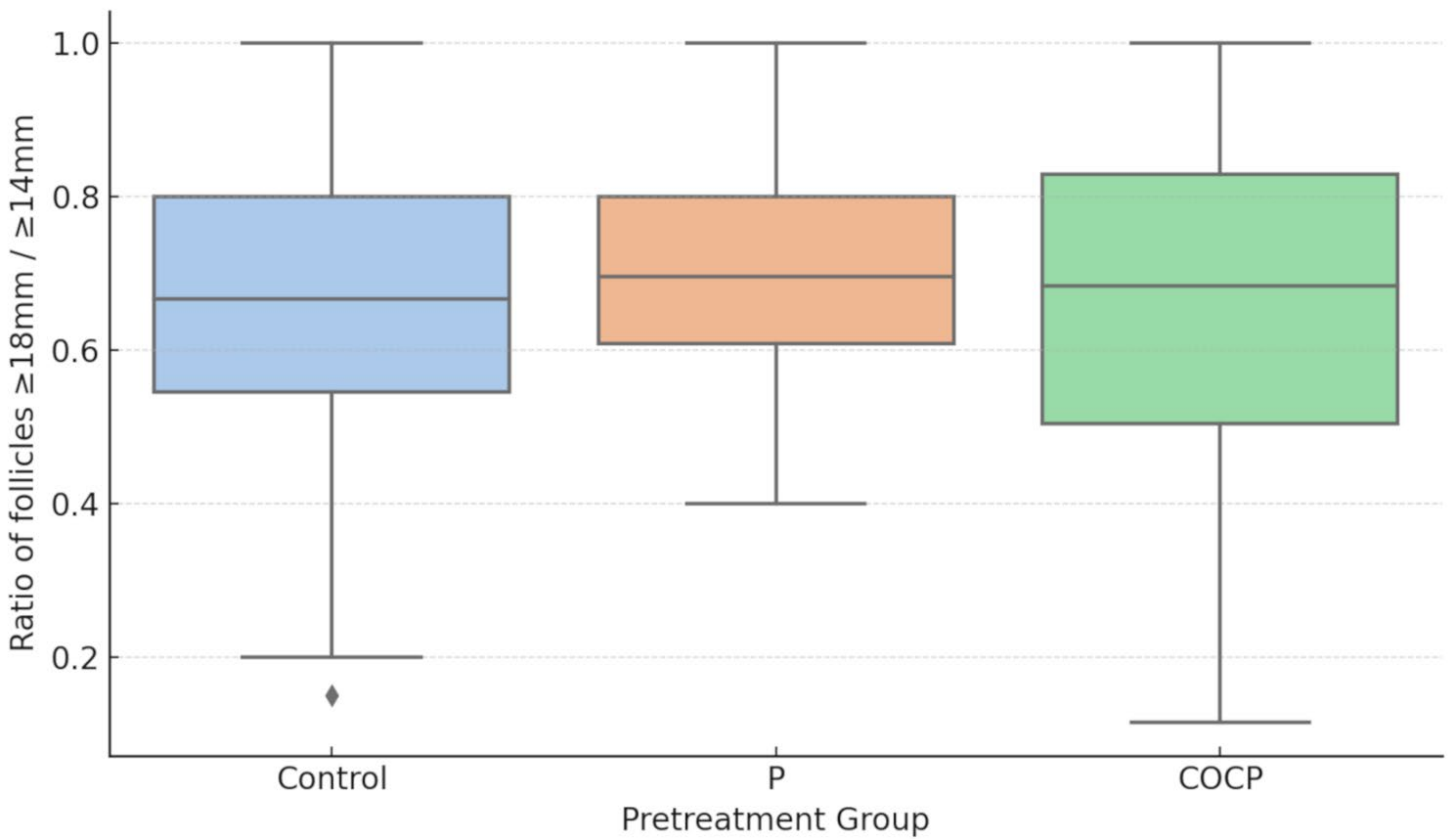
Boxplots demonstrate inter-group differences in variability

To visually assess differences in follicle synchronization, the 18 mm/14 mm ratio was plotted by group using boxplots (Fig. 2).

The P group demonstrated the least variability, as shown by the tight distribution and narrow interquartile range. In contrast, the COCP group exhibited the greatest variability, with a broader range and higher dispersion of individual values. These visual findings align with the statistical results from Levene’s test (*p* = 0.0001), further supported by post hoc analyses showing significant variance differences between all group pairs.

**Fig. 3:**
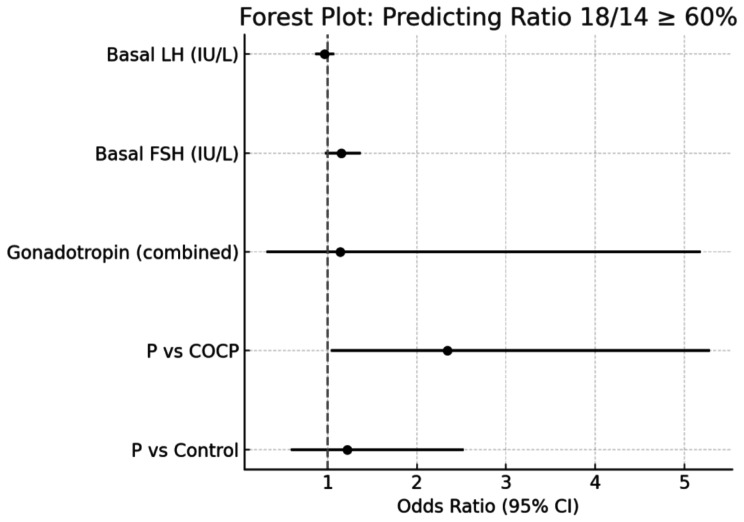
The analysis included pretreatment group (P vs. COCP, P vs. control), age, basal FSH, basal LH, and gonadotropin type

Logistic regression analysis was performed to identify factors associated with achieving a ratio of ≥ 60% for follicles ≥ 18 mm relative to those ≥ 14 mm (18/14 ratio). The independent variables included pretreatment group (P vs. COCP, P vs. control), basal FSH, basal LH, age, and gonadotropin type. The analysis revealed that the P group was significantly associated with a higher probability of achieving an 18/14 ratio ≥ 60%, compared to the COCP group (OR: 2.34, 95% CI: 1.04–5.28, *P* = 0.040). No significant difference was observed between the P group and the control group (OR: 1.22, 95% CI: 0.59–2.52, *P* = 0.59). (Fig. 3)

## Discussion

In this study, we evaluated the impact of different pretreatment strategies—P group, COCP group, and control group—on IVF/ICSI outcomes using a GnRH antagonist protocol. The results revealed that the P group showed a trend toward higher cumulative pregnancy and live birth rates compared with the other groups, along with significantly improved follicular synchronization. When using the ratio of follicles ≥ 18 mm to those ≥ 14 mm (18/14 ratio) as a proxy for synchrony, the P group exhibited the highest values and the lowest intra-group variability, suggesting a more uniform follicular cohort.

Additionally, the P group demonstrated significantly higher numbers of good-quality blastocysts and higher blastocyst development rates, indicating improved embryo developmental efficiency. These findings may be explained by the influence of progestin on endogenous hormonal dynamics. Progestin pretreatment has been shown to suppress GnRH pulsatility, thereby reducing FSH and LH secretion [13–15]. It also limits early follicular sensitivity to FSH, preventing premature recruitment and contributing to synchronized follicular development [16, 17].

Mechanistically, these effects may be mediated via progesterone’s action on hypothalamic kisspeptin neurons. Kisspeptin is a potent activator of the hypothalamic–pituitary–gonadal axis, stimulating GnRH secretion and, in turn, FSH and LH release [18–20]. Nearly all arcuate nucleus (ARC) kisspeptin neurons express progesterone receptors, and the surge in progesterone levels following ovulation suppresses LH secretion via these pathways [21, 22]. Furthermore, kisspeptin has been shown to inhibit the upregulation of FSH receptor (FSHR) expression in granulosa cells [23].

Thus, while LH surges are effectively prevented, FSH is not excessively suppressed; instead, follicular sensitivity to FSH is attenuated, suppressing early follicular recruitment prior to menses. This mechanism likely contributes to more synchronized follicle development at the onset of COS.

In contrast, COCPs contain ethinyl estradiol (EE), which acts to suppress endogenous gonadotropin release [24–26] and upregulate progesterone receptor expression, thereby potentiating progestin effects [27]. However, excessive suppression of endogenous gonadotropins with COCPs may delay stimulation onset, blunt E2 rise, and increase the duration and total dose of gonadotropins required [3].

Both P and COCP pretreatment may reduce the risk of functional ovarian cyst formation, a frequent issue in reproductive-aged women. In large cross-sectional studies, ovarian cysts larger than 30 mm were found in 4–7% of women undergoing baseline ultrasonography before hormonal contraception initiation [28]. These cysts have been associated with poor ovarian response in IVF cycles, particularly when measuring 30–60 mm [29–31]. Several studies have reported that pretreatment with oral contraceptives reduces the incidence of functional ovarian cysts [32], and this effect is particularly evident with progestins [33]. Therefore, prevention of cyst formation may be another contributing factor to improved follicular synchronization at the start of COS.

While some retrospective studies have associated hormonal contraceptive pretreatment with reduced live birth rates after fresh transfer (42.6% vs. 52.8%, *P* < 0.001) and lower cumulative live birth rates (62.8% vs. 67.6%, *P* = 0.01) [34], a 2017 Cochrane review of 29 RCTs in GnRH agonist and antagonist cycles found that pretreatment with hormonal contraception may reduce the risk of pregnancy loss [33]. However, other RCTs have shown no significant effect on clinical pregnancy or cumulative live birth rates when hormonal contraception was administered for 12–30 days with a 5-day washout period [35].

In our study, the P group showed significantly higher fertilization rates and blastocyst formation rates, which translated into an increased number of viable embryos per cycle. This enhanced oocyte-to-blastocyst efficiency likely contributed to the trend toward higher cumulative pregnancy and live birth rates in the P group. These results highlight the importance of optimizing follicular dynamics to improve embryological outcomes and overall ART success.

The superiority of the P group may also be linked to baseline LH levels. Persistently elevated LH during the follicular phase can trigger premature meiotic resumption, oocyte nuclear damage, and apoptosis [36, 37]. High LH also promotes excess androgen production by stimulating theca cells, leading to hyperandrogenism [38]. Elevated androgen concentrations in follicular fluid can disrupt intracellular calcium oscillations in oocytes, impeding cytoplasmic maturation and meiotic competence [39]. Oocytes retrieved under high LH conditions may exhibit excessive fragmentation and asymmetric cleavage, with negative implications for fertilization and embryo development [40].

In our study, the COCP group had significantly higher basal LH levels than the control and P groups. This may reflect the lower doses of progestin in COCP formulations (e.g., norgestrel 0.5 mg or levonorgestrel 0.09 mg), compared to the higher-dose norethisterone (5 mg) used in the P group [41]. Therefore, the P group may have benefited from stronger suppression of LH and androgens, resulting in the retrieval of higher-quality oocytes and better embryo development.

Taken together, our findings suggest that progestin-only pretreatment leads to superior follicular synchronization, lower LH exposure, and improved embryo developmental potential. These advantages may ultimately enhance ART efficiency, particularly by increasing the number of transferable embryos and improving the likelihood of achieving pregnancy and live birth per cycle.

## Data Availability

The datasets supporting the findings of this study are available from the corresponding author upon reasonable request.

## Abbreviations

AFC: Antral Follicle Count
AMH: Anti-Müllerian Hormone
ANOVA: Analysis of Variance
ARC: Arcuate Nucleus
ART: Assisted Reproductive Technology
CLBR: Cumulative Live Birth Rate
COCP: Combined Oral Contraceptive Pills
COS: Controlled Ovarian Stimulation
E2: Estradiol
EE: Ethinyl Estradiol
ET: Embryo Transfer
FSH: Follicle-Stimulating Hormone
FSHR: Follicle-Stimulating Hormone Receptor
GnRH: Gonadotropin-Releasing Hormone
HMG: Human Menopausal Gonadotropin
HPO: Hypothalamic–Pituitary–Ovarian
HRC: Hormone Replacement Cycle
ICSI: Intracytoplasmic Sperm Injection
IQR: Interquartile Range
IVF: In Vitro Fertilization
LH: Luteinizing Hormone
MII: Metaphase II Oocyte
NC: Natural Cycle
OR: Odds Ratio
RCT: Randomized Controlled Trial
SD: Standard Deviation
SET: Single Embryo Transfer
rFSH: Recombinant Follicle-Stimulating Hormone
